# Using STR Data to Investigate the Impact of the Studbook Cap on Genetic Diversity in the American Standardbred Horse from 1998 to 2021

**DOI:** 10.3390/genes16070748

**Published:** 2025-06-27

**Authors:** Felipe Avila, Elizabeth Esdaile, Rebecca R. Bellone

**Affiliations:** 1 Veterinary Genetics Laboratory, School of Veterinary Medicine, University of California–Davis, Davis, CA 95616, USA; ffavila@ucdavis.edu (F.A.); esesdaile@ucdavis.edu (E.E.); 2 Department of Population Health and Reproduction, School of Veterinary Medicine, University of California–Davis, Davis, CA 95616, USA

**Keywords:** microsatellites, equine, inbreeding, population genetics, Standardbreds

## Abstract

**Background/Objectives:** Standardbreds, a breed of horses used in harness racing at either the trot or the pace, established a closed studbook in 1973. Concerns about genetic diversity within the breed led the United States Trotting Association (USTA) to establish a limit of mares bred per stallion (i.e., a studbook cap) in 2009. Here, we aimed to evaluate the impact of the breeding restrictions on genetic diversity between and among subpopulations. **Methods**: Sixteen short tandem repeats (STRs) were analyzed across a dataset of 176,424 Standardbreds foaled in the United States between 1998 and 2021. We examined allelic richness (*Na*), number of effective alleles (*Ne*), expected heterozygosity (*H_E_*), observed heterozygosity (*H_O_*), inbreeding coefficient (*F_IS_*), and fixation index (*F_ST_*) across 24 years, differentiating by gate type, and comparing pre-(1998–2009) and post-(2010–2021) studbook cap periods using regression analysis. **Results**: Our results support decreased genetic diversity for both trotters and pacers over time. However, pacing Standardbreds exhibited significantly slower rates of decrease in genetic diversity after the 2009 studbook cap, as evidenced by *Ne*, *H_E_*, and *F_IS_* (*P_Bonferroni_* < 0.01). Additionally, moderate levels of genetic differentiation were found between trotters and pacers (0.05 < *F_ST_* < 0.09), which increased over time. **Conclusions**: Given that the rate of loss of diversity does not appear to differ pre and post studbook cap in trotters and that there is an increase in genetic differentiation between the groups over time, developing additional breeding tools and strategies is necessary to help the subpopulation mitigate further decline.

## 1. Introduction

American Standardbreds are a harness racing horse breed that performs at either a trot (a diagonally opposed and symmetrical two-beat gait) or pace (a laterally symmetrical two-beat gait). This breed of horse has had a closed studbook since 1973, and since crossbreeding is not allowed, concerns of genetic diversity have been considered in several studies starting shortly after the studbook was closed. Specifically, studies in the 1980s and 1990s were performed utilizing blood markers, short tandem repeats (STRs), and pedigree records to investigate genetic diversity in this breed. These historical studies showed that trotters were more inbred than pacers and that pacers had higher conception and foaling rates than trotters, likely due to higher expected heterozygosity levels noted [1,2]. Most importantly, however, these studies reported that the mean observed heterozygosity estimates for both trotters and pacers were decreasing over time [1,2,3,4,5]. The aforementioned data, the decrease in observed heterozygosity, and the concern for potential health risks due to inbreeding spurred the United States Trotting Association (USTA)—the governing body of American Standardbreds—to impose a studbook cap (i.e., restrict the number of mares bred by a stallion per year) to help maintain genetic diversity in the breed. Starting in 2009, the USTA imposed a studbook limit of 140 mares bred per year for trotting stallions debuting in 2009 and on. Similarly, a studbook limit of 160 mares bred per year was imposed on pacing sires debuting in 2009, decreasing to 150 in 2010 and 140 after 2011. More than fifteen years after its establishment, the potential effect of the studbook cap on mitigating the loss of genetic diversity in trotting and pacing Standardbreds has yet to be fully assessed.

Further, since the 1990s, reported measures of heterozygosity and genetic diversity in the Standardbred have only been evaluated in two additional studies. In a study published in 2013, Petersen and colleagues utilized SNP genotypes from the Illumina SNP50 Beadchip from 40 Standardbreds born in the United States and Norway to ascertain genetic diversity in the breed [6]. That dataset included 32 trotters and eight pacers. The authors observed low levels of genetic diversity in Standardbreds as measured by expected heterozygosity (*H_E_*) values (0.270 < *H_E_* < 0.300), determined based on four different SNP sets pruned based upon varying LD levels. Moreover, the authors observed relatively high levels of genetic differentiation between US- and Norway-born Standardbreds (*F_ST_* = 0.020) when compared to those estimated for the dataset at large, which comprised over one thousand horses from 38 different breeds [6]. Finally, in that study, the authors observed a significant excess of homozygosity in the United States Standardbred population (*F_IS_* = 0.039) but not in the Norwegian population (*F_I_*_S_ = −0.004). The authors hypothesized that this was due to US Standardbreds being divergently selected for racing at either the pace or the trot, thus creating structure within the breed in the United States [6].

More recently, we utilized genotypes from 16 STRs (also called microsatellites) from a total of 50,621 trotting and pacing Standardbreds, including foals born between 2010 and 2015 plus their sires and dams, to investigate trends in genetic diversity within this half-generation interval immediately post the studbook cap [7]. Results showed that both trotting and pacing sires were significantly less genetically diverse than dams based on expected heterozygosity estimates. Trotting and pacing sires also showed significantly lower allelic richness than their offspring, and pacing offspring were significantly less diverse than their dams based on expected heterozygosity values. However, inbreeding coefficient (*F_IS_*) values below zero were observed in both Standardbred subpopulations for the 6-year period investigated, thus suggesting that breeding practices and, in particular, the dams maintained genetic diversity in both subpopulations [7]. However, only a half-generation interval after the studbook cap (2010–2015) has been investigated; therefore, evaluating a larger time scale that encompasses both pre-and post-cap genetic data is necessary to assess the studbook cap’s impact on genetic diversity.

In this study, we aimed to investigate trends in genetic diversity in American Standardbred horses using genotypes from 16 STR markers across the entire population of trotting and pacing Standardbreds registered in the United States between 1998 and 2021. Our overarching goal was to utilize these longitudinal data spanning 24 years to assess the potential impact of the 2009 studbook cap on indices of genetic diversity in both trotting and pacing Standardbreds. Finally, we also aimed to evaluate how the sire book size impacted genetic diversity over the 24-year period.

## 2. Materials and Methods

### 2.1. Data Collection

To assess genetic diversity, genotyping data for all registered Standardbred foals born between 1998 and 2021 were obtained from Bureau Veritas Laboratories, which performs STR genotyping as part of the mandatory parentage verification that is required for a Standardbred to be registered with the USTA. Genotypes from 16 STR markers were provided: 12 belonging to the core panel recommended by the International Society of Animal Genetics (ISAG) for equine parentage verification (AHT4, AHT5, ASB2, ASB17, ASB23, HMS2, HMS3, HMS6, HMS7, HTG4, HTG10, and VHL20) and 4 from the backup panel of ISAG equine parentage verification markers (CA425, HMS1, HTG7, and LEX33) [8,9].

### 2.2. Data Analysis

Horses were grouped according to year of birth, country of birth, and gait. Records were obtained from 106,355 trotting and 153,853 pacing Standardbreds, for a total of 260,208 horses. However, we restricted the analysis to foals with STR profiles for all 16 markers used in this study and born in the United States, since the studbook cap was only applied to this country. After filtering for STR data completeness, 101,283 trotters and 142,826 pacers remained in the dataset. Further filtering for country of birth removed 26,132 trotting and 57,652 pacing individuals from the analysis. The final dataset comprised 80,223 trotters and 96,201 pacers (total = 176,424 individuals, Table 1). Pedigree records were provided by the USTA, and the gait phenotype for each horse (trotter or pacer) was established based on performance records, also provided by the USTA. When no performance records were available for a given horse, they were assigned the gait of their sire (Supplemental Table S1). In order to investigate trends in genetic diversity over time and the impact of the 2009 studbook cap within gait types, foal crops were divided based on the 12 years before the studbook cap (1998–2009) and the 12 years after the cap (2010–2021), as well as in half-generation intervals (6 years) spanning the study period. Additionally, for each year, the horses were divided into 3 groups based on the total number of offspring sired by each stallion for the 24 years spanned by this study. Groupings were divided based on approximately 33% of the total offspring and categorized based on the number of foals produced by each sire, as previously described [7]. Briefly, offspring of the highest producing sires were classified as “high-book”; individuals produced by the second most prolific group of stallions were categorized as “mid-book”; and the term “low-book” was applied to the resulting offspring by the lowest producing sires for each year.

The Microsoft Excel add-in GenAlex v.6.5 was used to calculate the number of alleles (*Na*), number of effective alleles (*Ne*), expected heterozygosity (*H_E_*), observed heterozygosity (*H_O_*), and inbreeding coefficient (*F_IS_*) for each STR marker [10]. Then, the mean values for each diversity parameter were calculated by averaging the values of each locus. A pairwise fixation index (*F_ST_*) using 20,000 permutations [11] was also calculated between trotters and pacers for each year using the R v4.2.1 package hierfstat [12]. *T*-test comparisons of *Na*, *Ne*, *H_E_*, *H_O_*, and *F_IS_* between years and between the pre- and post-studbook cap time periods, as well as linear regression models (lm) of each diversity parameter between the pre- and post-studbook cap time periods, were calculated using R version 4.2.1 [13]. A Bonferroni-corrected significance level of *P* < 0.05 was used to determine *t*-test significance (*P_Bonferroni_* =1 − (1 − x)^n^). The R package ‘emmeans’ version 1.8 [14] was used to determine the statistical difference in slopes of regression lines between the 12-year time periods before and after the studbook cap (1998–2009 and 2010–2021), as well as for comparing half-generation time intervals: 1998–2003, 2004–2009, 2010–2015, and 2016–2021 (*P* < 0.05 for all tests).

## 3. Results

### 3.1. Allelic Richness (Na)

In trotting Standardbreds, the mean *Na* value for the 12 years preceding the studbook cap (1998–2009) was estimated at 7.67, whereas a mean *Na* of 7.36 was estimated for the post-studbook cap period (2010–2021). This value for the period after the cap was statistically significantly lower even after multiple-test correction (*P_Bonferroni_* < 0.01) (Table 2, Supplemental Table S2). When grouped by book size, *Na* values for each of the three trotting groups followed the same trend as the population at large, with lower average post-studbook cap *Na* values when compared to pre-studbook values. However, the difference was not statistically significant for high-book offspring (6.84 vs. 6.75; *P_Bonferroni_* = 0.14). Conversely, the mean number of alleles was significantly lower for mid-book offspring (7.02 vs. 6.86; *P_Bonferroni_* = 0.02) and for the low-book foal crops (7.35 vs. 7.04; *P_Bonferroni_* < 0.05) in the 12 years following the studbook cap when compared to the pre-cap period (Supplemental Table S3). Regression analysis showed that the rate of change in *Na* was only significantly different (*P* < 0.05) between two periods prior to the studbook cap in trotters: 1998–2003 (slope = 0.079) vs. 2004–2009 (slope = −0.084) (Supplemental Table S5).

In the pacing subpopulation, the mean *Na* was significantly lower after the studbook cap when compared to the pre-studbook average (*P_Bonferroni_* < 0.05) (Table 2 and Supplemental Table S2). When split based on book size, mean *Na* values were statistically significantly lower post studbook cap when compared to pre-cap periods for all three book size comparisons: high-book (7.28 vs. 6.7; *P_Bonferroni_* < 0.05), mid-book (7.44 vs. 6.87; *P_Bonferroni_* < 0.05), and low-book offspring (7.70 vs. 7.21; *P_Bonferroni_* < 0.05) (Supplemental Table S4). The regression analysis of *Na* values conducted over time did not support any statistically significant difference in the rate of change pre and post the studbook cap (Supplemental Table S5).

### 3.2. Effective Number of Alleles (Ne)

*Ne* values were significantly lower post studbook cap for both trotting (3.47 vs. 3.30, *P_Bonferroni_* < 0.05) and pacing Standardbreds (3.44 vs. 3.21, *P_Bonferroni_* < 0.05) (Table 2 and Supplemental Table S2). When both populations were divided by book size, mean *Ne* values were statistically significantly lower (*P_Bonferroni_* < 0.05) post studbook cap when compared to the pre-cap period for all book sizes (high, medium, and low) (Supplemental Tables S3 and S4). Slopes of the regression lines were not statistically significantly different between any time periods evaluated in the trotting subpopulation. In pacers, however, the slopes of the regression lines were significantly different (*P* < 0.05) between the two periods preceding the cap, 1998–2003 (slope = −0.031) and 2004–2009 (slope = −0.017; *P* = 0.017), as well as between the 12 years preceding the cap (1998–2009; slope = −0.024) and the 12 years after the cap (2010–2021; slope = −0.009; *P* < 0.01) (Supplemental Table S5).

### 3.3. Expected Heterozygosity (H_E_)

The mean post-cap *H_E_* value (0.66) for trotters was significantly lower than that estimated pre-cap average (0.68; *P_Bonferroni_* < 0.05) (Table 2 and Supplemental Table S2). Similarly, average *H_E_* values estimated for all three trotting groups based on book size were statistically significantly lower (*P_Bonferroni_* < 0.05) after the 2009 studbook cap when compared to the pre-cap period (Supplemental Table S3). The regression analysis of *H_E_* values for trotters across half-generation intervals supports the rate of change being significantly different (*P* < 0.05) between the two time periods following the studbook cap, 2010–2015 (slope = −0.002) vs. 2016–2021 (slope = −0.0005; *P* < 0.05), but not between pre- and post-cap time periods (Supplemental Table S5).

In pacing Standardbreds, similar trends were observed for *H_E_* across the study period. The mean *H_E_* value post studbook cap (0.66) was significantly lower than that of the pre-cap period (0.68; *P_Bonferroni_* < 0.05) (Table 2 and Supplemental Table S2). Again, similarly to the trotting subpopulation, mean *H_E_* values estimated for high, medium, and low book sizes in pacing Standardbreds were all statistically significantly lower post cap when compared to pre-cap counterparts (*P_Bonferroni_* < 0.05; Supplemental Table S4). Further, the regression analysis of *H_E_* in pacing Standardbreds supports the rate of decrease in expected heterozygosity values being significantly slower for 2010–2021 (slope = −0.001) compared to the pre-studbook cap time period (1998–2009, slope = −0.003, *P* < 0.01 (Supplemental Table S5).

### 3.4. Observed Heterozygosity (H_O_)

In trotters, the mean *H_O_* value estimated pre-studbook limit (0.68) was significantly higher than that estimated for the post-cap period (0.667; *P_Bonferroni_* < 0.05) (Table 2 and Supplemental Table S2). The regression analysis of mean *H_O_* values did not show a statistical difference in the slopes of regression lines between any of the periods included in this study (Supplemental Table S5). When the trotting offspring was divided into book sizes, statistical analysis showed that the mean *H_O_* values were significantly lower (*P_Bonferroni_* < 0.05) after the studbook cap for all three book sizes when compared to the 12 years before the cap (Supplemental Table S3). 

A similar scenario was observed for *H_O_* for the pacing subpopulation, in which the mean post-cap *H_O_* value (0.67) was significantly lower (*P_Bonferroni_* < 0.05) than that estimated for the pre-studbook cap period (0.69; Supplemental Table S2). Similarly to trotters, *H_O_* values in the post-studbook cap period were significantly lower than those estimated before the cap for high book, medium book, and low book pacing offspring groups (*P_Bonferroni_* < 0.05; Supplemental Table S4). Additionally, regression analysis did not show a significant difference in the slopes of the regression lines of *H_O_* values for pacers between any of the study periods (Supplemental Table S5).

### 3.5. Inbreeding Coefficient (F_IS_)

For trotters, all *F_IS_* values were negative across all years, with no statistical differences noted by year or by twelve-year pre- and twelve-year post-studbook cap time period comparisons (Table 2 and Supplemental Table S2). When considering half-generation intervals, a statistically significant difference was observed in the slopes of the regression lines of mean *F_IS_* values in trotters between the 2004–2009 (slope = 0.0002) and the 2010–2015 intervals (slope = −0.001, *P_Bonferroni_* < 0.05), but not between other time periods (Supplemental Table S5). However, year and *F_IS_* were not correlated (*R*^2^ = 0.14) in trotters. When comparing different book sizes, high-book trotting offspring had significantly lower mean *F_IS_* values (−0.03) post studbook cap when compared to the pre-cap average (−0.02) (*P_Bonferroni_* < 0.05, Supplemental Table S3). Mean *F_IS_* values for medium- and low-book offspring groups were not statistically significantly different before and after the cap.

Contrary to the trotting subpopulation, in which negative mean *F_IS_* values were estimated for all 24 years included in this study, positive *F_IS_* values were estimated for the first six years (1998–2003) in pacing Standardbreds, whereas in all remaining years *F_IS_* values were negative (Table 2). Mean *F_IS_* values were significantly lower (*P_Bonferroni_* < 0.05) post studbook cap for all three book size groups (high, medium, and low book) when compared to pre-cap averages for pacers (Supplemental Table S4). Finally, investigating differences before and after the studbook cap shows that the rate of change in pacers was greater before the studbook cap, as evidenced by the slope of the regression line for 1998–2009 (slope = −0.002) compared to 2010–2021 (slope = −0.0001; *P* < 0.05, *R*^2^ = 0.60) (Supplemental Table S5).

### 3.6. Pairwise Fixation Index (F_ST_)

**Figure 1:**
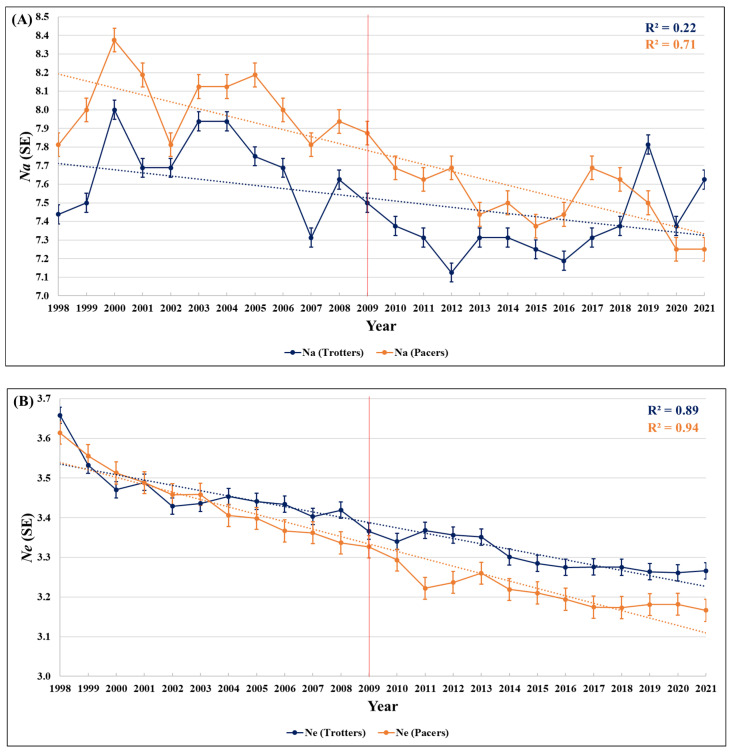

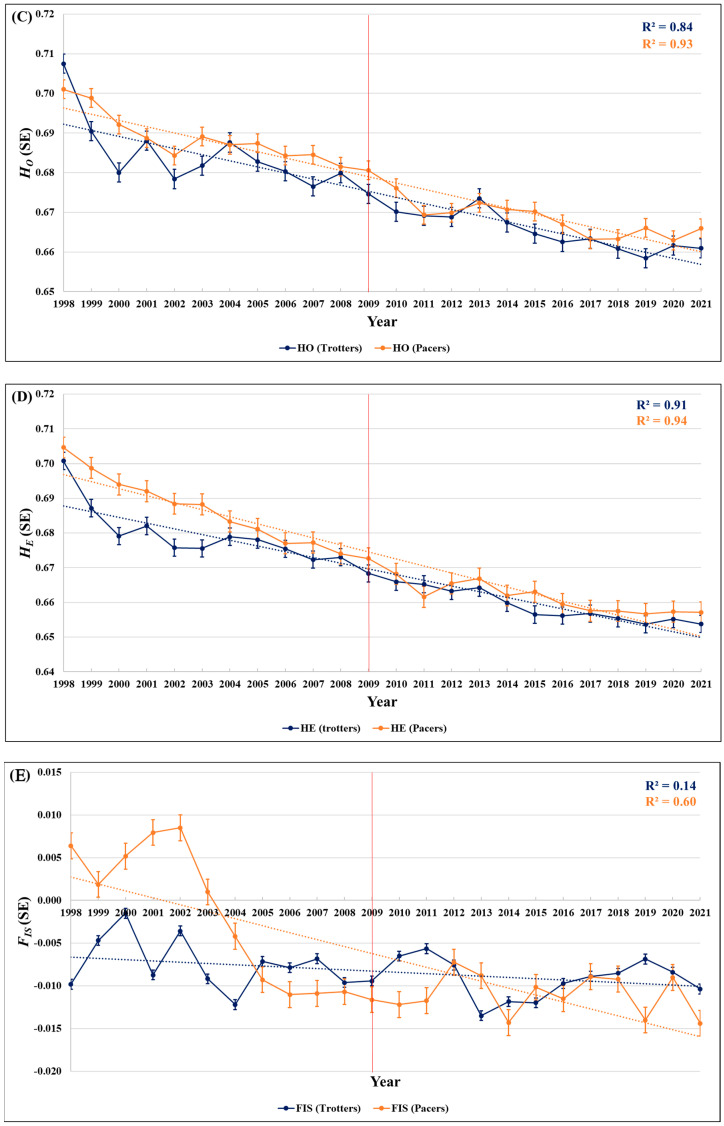

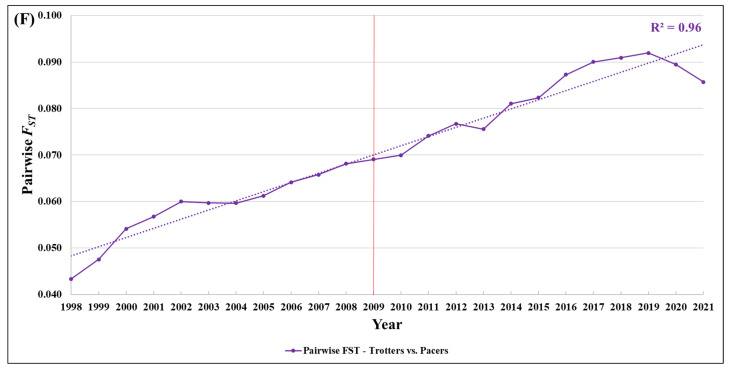
Trends in diversity measures of trotting (blue) and pacing (orange) Standardbred horses born in the United States between 1998 and 2021. Presented are (**A**) allelic richness (*Na*); (**B**) number of effective alleles (*Ne*); (**C**) expected heterozygosity *(H_E_*); (**D**) observed heterozygosity (*H_O_*); (**E**) inbreeding coefficient (*F_IS_*); and (**F**) pairwise fixation index (*F_ST_*). *Ne*, *H_E_*, and *H_O_* showed downward trends across the study period, supporting an overall loss of diversity within the breed and subpopulations. A significant difference was observed in the slopes of the regression lines between pre- and post-studbook cap time periods for *Ne*, *H_E_*, and *F_IS_* (*P* < 0.01) in pacers. Similarly, the slopes of *H_E_* regression lines were statistically significantly different in trotters but only for the two half-generation time intervals after the studbook cap. Pairwise *F_ST_* values between trotters and pacers trended upwards, suggesting increasing genetic differentiation between these subpopulations across the 24-year period evaluated. Dashed lines represent the regression trend lines for each graph, and the red vertical line represents the year (2009) when the studbook cap was first imposed by the USTA.

Moderate levels of genetic differentiation (0.05 < *F_ST_* < 0.09) [11,15] were found between trotters and pacers across the study period (Supplemental Table S6). Within the trotting and pacing subpopulations, pairwise *F_ST_* values below 0.05 were calculated for all intergroup comparisons, indicating that little to no genetic differentiation occurred when grouping by book size within gait (Supplemental Table S7). Results for the diversity metrics are summarized in Figure 1.

## 4. Discussion

In this study, genotypes for 16 STRs (12 from the ISAG equine parentage verification core panel and four from the backup panel) were utilized to investigate genetic diversity in nearly the entire population of trotting and pacing Standardbreds born in the United States and registered with the USTA between 1998 and 2021 (*n* = 176,424). Additionally, the potential impact of the 2009 studbook cap in mitigating loss of diversity in the breed was investigated. To our knowledge, this is the first longitudinal study to utilize microsatellite genotypes generated from parentage testing for registration purposes from close to the entire population of a pure breed of horses to evaluate genetic diversity.

STRs were first reported as a potential replacement for blood typing in horse paternity testing in the early 1990s [16,17,18] and validated later that decade [19]. Since then, equine breed associations that require parentage verification for registration purposes have relied on STRs for parentage testing. Because of this requirement, the data presented in this study represent a genetic snapshot of nearly every Standardbred foaled in the United States and registered with the USTA over a 24-year period. Additionally, STRs represent an inexpensive, robust, and reliable tool to investigate population genetic parameters due to their highly polymorphic nature and the requirement of relatively small quantities of low-quality input DNA for accurate genotyping. Therefore, these markers have been successfully used in numerous studies aimed at investigating trends in genetic diversity in various species for decades, including in horses [20,21,22,23,24,25].

Recently, we reported genetic diversity indices calculated using genotypes from the same panel of 16 STRs used in this study, derived from horses foaled from 2010 to 2015 (i.e., immediately following the studbook cap) and their sires and dams (*n* = 50,621) [7]. Our results indicated no significant change in *H_E_* or any other measured diversity metric across the 6 years of foal crops examined, as well as no significant differences in the amount of inbreeding between the trotting and pacing American Standardbreds based on *F_IS_* values. Finally, a moderate level of genetic differentiation was calculated between pacers and trotters born between 2010 and 2015 in that study, with pairwise *F_ST_* values ranging from 0.080 to 0.084 [7].

In this study, we aimed to use the genetic diversity results for the 2010–2015 foal crops from our previous report as a baseline in order to assess trends in genetic diversity in Standardbreds over a 24-year period and investigate the impact of the 2009 studbook cap in mitigating the loss of genetic diversity in this breed. While in the previous period of six years a loss of genetic diversity was not observed over time, this longer longitudinal assessment allowed us to observe a loss of diversity in Standardbreds across all metrics evaluated and make pre- and post-studbook cap comparisons. Specifically, significantly slower rates of decrease in genetic diversity were found in pacing Standardbreds after the 2009 studbook cap, as evidenced by *Ne*, *H_E_*, and *F_IS_* (*P_Bonferroni_* < 0.01) (Figure 1, Supplemental Table S5). Taken together, these data support that, for pacers, either the studbook cap had an impact in slowing the rate of genetic diversity loss or that there was a change in breeding practices helping maintain heterozygosity since the cap (or a combination of the two). These findings were corroborated by statistically significantly lower mean *F_IS_* values for all three book size groups (high, medium, and low) of pacing offspring post the studbook cap when compared to pre-cap averages (Supplemental Table S4) and the finding in our previous study that mares may be maintaining diversity in pacing Standardbreds [7]. A follow-up study using mitochondrial DNA (mtDNA) data to investigate the maternal contribution to genetic diversity in the breed is warranted.

In trotters, while a pre-cap vs. post-cap difference in the rate of loss of diversity was not detected, the slopes of the regression analysis of *H_E_* for the two half-generation intervals after the studbook cap were significantly different, with 2010–2015 being greater than 2016–2021. This suggests that the studbook cap may have helped slow the loss of heterozygosity in trotters, but its impact is taking longer to be detected given the higher levels of inbreeding in trotters prior to the studbook cap when compared to pacers [1,2] (Figure 1, Supplemental Table S5). However, this finding should be interpreted with caution, as other diversity measures do not indicate a potential reduction in the rate of loss of genetic diversity in trotters. Therefore, continuing to monitor this trend is warranted to further understand the impact of breeding restrictions on diversity loss in trotting Standardbreds.

Trends in *Na* values further highlight the divergence between trotters and pacers, where both gait types showed significant post-cap reductions, but pacers maintained relatively higher *Na* values, particularly among high- and mid-book sires (Supplemental Tables S3 and S4). Conversely, trotters experienced a more uniform decrease across all book sizes, again suggesting that the cap had less of an effect on mitigating diversity loss within this subpopulation.

In our previous study, we estimated moderate levels of genetic differentiation (0.080 < *FST* < 0.084) [15] when comparing the data from the same panel of STRs in pacing and trotting Standardbreds foaled between 2010 and 2015 [7]. Here, similar results were obtained for these foal crops (Figure 1, Supplemental Tables S6 and S7). While the trends remain the same, the values are slightly different, mainly due to two reasons: in our previous study, foal crops were not filtered by country and thus comprised horses born worldwide; and the number of offspring used in this study was larger. Interestingly, *F_ST_* values for the whole 24 years of foal crops included in this study provided a more global picture of genetic differentiation based on gait within Standardbreds. Since 1998 (*F_ST_* = 0.043), the level of genetic differentiation between pacers and trotters has been increasing, with 2019 showing the highest pairwise *F_ST_* between subpopulations (*F_ST_* = 0.092), followed by a slight decrease in the two subsequent years (Figure 1, Supplemental Table S6). This differentiation is likely due to differences in breeding practices, selection pressures, and limited gene flow between these subpopulations, with gene flow occurring mostly from trotting to pacing lines [4]. This flow of alleles from trotters to pacers might also help mitigate the loss of genetic diversity noted in the pacing subpopulation.

In conclusion, this study evaluated genetic diversity metrics for the US populations of trotting and pacing Standardbreds comprising 176,424 horses foaled between 1998 and 2021. These data constitute, to our knowledge, the longest (24 years) longitudinal study to utilize STR genotypes from an entire population of registered horses to investigate diversity trends in a breed. These data suggest that the implementation of the studbook cap may have helped mitigate the loss of diversity in the pacing Standardbred; in trotters, further analysis over a longer post-studbook cap time span is warranted. Overall, the decline of all genetic diversity measures in both subpopulations highlights the need to continue to monitor breeding practices and develop tools and strategies to mitigate further loss of heterozygosity in this breed. While STRs provide a snapshot of haplotypes from 16 loci in the genome, they do not provide a complete genomic picture. Thus, additional work aimed at identifying genome-wide haplotypes is warranted, in order to detect minor haplotypes and/or alleles in the population to target and maintain diversity in this breed. The results serve as a foundation for continued monitoring of the impact of the studbook cap and diversity levels of the American Standardbred.

## Figures and Tables

**Table 1:** Number of registered trotting and pacing Standardbreds foaled in the USA with genotypes for all 16 STR markers used in this study per year.

Year	Trotters	Pacers
1998	1145	2188
1999	2357	3882
2000	3785	5862
2001	3832	5866
2002	3835	5737
2003	3850	5907
2004	3986	5255
2005	3880	5300
2006	3915	4993
2007	3512	3640
2008	3540	4360
2009	3408	3908
2010	3344	4091
2011	3070	3830
2012	3100	3580
2013	2908	3470
2014	2971	3489
2015	3087	3211
2016	3033	3036
2017	3195	3014
2018	3512	2986
2019	3821	3049
2020	4073	3104
2021	3064	2443
**Total**	**80,223**	**96,201**

**Table 2:** Measures of diversity in trotting and pacing Standardbreds born in the United States in the 12-year period before the studbook cap (1998–2009) and the 12-year period after the cap (2010–2021). Allelic richness (*Na*), number of effective alleles (*Ne*), observed and expected heterozygosity (*H_O_* and *H_E_*, respectively), and inbreeding coefficient (*F_IS_*) are shown. Significant *P*-values between pre- and post-studbook cap means for each diversity measure are bolded.

Trotters					
	*Na*	*Ne*	*H_O_*	*H_E_*	*F_IS_*
**Pre-studbook cap (1998–2009) mean**	7.67	3.46	0.68	0.68	−0.01
**Post-studbook cap (2010–2021) mean**	7.36	3.30	0.67	0.66	−0.01
***P*-value**	**1 × 10^−3^**	**<1 × 10^−5^**	**1 × 10^−4^**	**1 × 10^−4^**	1.3 × 10^−1^
**Pacers**					
	***Na***	***Ne***	***H_O_***	***H_E_***	***F_IS_***
**Pre-studbook cap (1998–2009) mean**	8.02	3.44	0.69	0.69	0.00
**Post-studbook cap (2010–2021) mean**	7.51	3.21	0.67	0.66	−0.01
***P*-value**	**<1 × 10^−5^**	**<1 × 10^−5^**	**<1 × 10^−5^**	**<1 × 10^−5^**	**2.1 × 10^−3^**

## Data Availability

These data will be made available upon reasonable request to the corresponding author.

## Supplementary Materials

The following supporting information can be downloaded at: https://www.mdpi.com/article/10.3390/genes16070748/s1, Table S1: Number of pacers and trotters for which gait was assigned based on the individual's performance records or the sire's gait in the case of horses with no racing history. Table S2: Genetic diversity measures in trotting and pacing Standardbreds foaled in the United States between 1998 and 2021. Number of individuals analyzed per year (*N*), Allelic richness (*Na*), number of effective alleles (*Ne*), observed and expected heterozygosity (*H_O_* and *H_E_*, respectively), and inbreeding coefficient (*F_IS_*) are shown. SE = standard error of the mean. Table S3: Statistical analysis of measures of genetic diversity in trotting Standardbreds of different book sizes (high, medium, and low) foaled in the United States between 1998 and 2021. Number of individuals analyzed per year (*N*), allelic richness (*Na*), number of effective alleles (*Ne*), observed and expected heterozygosity (*H_O_* and *H_E_*, respectively), and inbreeding coefficient (*F_IS_*) are shown. Significant *P*-values between pre- and post-studbook cap means for each diversity measure are bolded. Table S4: Statistical analysis of measures of genetic diversity in pacing Standardbreds of different book sizes (high, medium, and low) foaled in the United States between 1998 and 2021. Number of individuals analyzed per year (*N*), allelic richness (*Na*), number of effective alleles (*Ne*), observed and expected heterozygosity (*H_O_* and *H_E_*, respectively), and inbreeding coefficient (*F_IS_*) are shown. Significant *P*-values between pre- and post-studbook cap means for each diversity measure are bolded. Table S5: Slopes of regression lines between time periods for trotting and pacing Standardbred foal crops. *P*-values of the difference in slopes of the regression are listed below each pair of slopes. Bolded *P*-values are significant at *P* < 0.05. Table S6: Pairwise *F_ST_* values between trotting and pacing Standardbreds from 1998 to 2021. Table S7: Pairwise *F_ST_* values between trotting and pacing Standardbreds foaled in the United States between 1998 and 2021. *F_ST_* values between each subpopulation per year are shown below the diagonal.
